# Endurance exercise has a negative impact on the onset of SOD1-G93A ALS in female mice and affects the entire skeletal muscle-motor neuron axis

**DOI:** 10.3389/fphar.2024.1360099

**Published:** 2024-03-25

**Authors:** Silvia Scaricamazza, Valentina Nesci, Illari Salvatori, Gianmarco Fenili, Marco Rosina, Michela Gloriani, Maria Paola Paronetto, Luca Madaro, Alberto Ferri, Cristiana Valle

**Affiliations:** ^1^ IRCCS Fondazione Santa Lucia, Rome, Italy; ^2^ Department of Systems Medicine, University of Roma “Tor Vergata”, Rome, Italy; ^3^ Department of Experimental Medicine, University of Roma “La Sapienza”, Rome, Italy; ^4^ Department of Movement, Human and Health Sciences, University of Rome “Foro Italico”, Rome, Italy; ^5^ Neurology Unit, PTV Foundation Tor Vergata University Hospital, Rome, Italy; ^6^ Department of Anatomy, Histology, Forensic Medicine and Orthopedics, University of Roma “La Sapienza”, Rome, Italy; ^7^ Laboratory Affiliated to Istituto Pasteur Italia-Fondazione Cenci Bolognetti, Rome, Italy; ^8^ National Research Council (CNR), Institute of Translational Pharmacology (IFT), Rome, Italy

**Keywords:** amyotrophic lateral sclerosis, physical activity, endurance exercise, neurodegeneration, neuroinflammation, SOD1-G93A mice

## Abstract

**Background:**

Amyotrophic lateral sclerosis (ALS) is a fatal neuromuscular disease characterized by the degeneration of motor neurons that leads to muscle wasting and atrophy. Epidemiological and experimental evidence suggests a causal relationship between ALS and physical activity (PA). However, the impact of PA on motor neuron loss and sarcopenia is still debated, probably because of the heterogeneity and intensities of the proposed exercises. With this study, we aimed to clarify the effect of intense endurance exercise on the onset and progression of ALS in the SOD1-G93A mouse model.

**Methods:**

We randomly selected four groups of twelve 35-day-old female mice. SOD1-G93A and WT mice underwent intense endurance training on a motorized treadmill for 8 weeks, 5 days a week. During the training, we measured muscle strength, weight, and motor skills and compared them with the corresponding sedentary groups to define the disease onset. At the end of the eighth week, we analyzed the skeletal muscle-motor neuron axis by histological and molecular techniques.

**Results:**

Intense endurance exercise anticipates the onset of the disease by 1 week (age of the onset: trained SOD1-G93A = 63.17 ± 2.25 days old; sedentary SOD1-G93A = 70.75 ± 2.45 days old). In SOD1-G93A mice, intense endurance exercise hastens the muscular switch to a more oxidative phenotype and worsens the denervation process by dismantling neuromuscular junctions in the tibialis anterior, enhancing the Wallerian degeneration in the sciatic nerve, and promoting motor neuron loss in the spinal cord. The training exacerbates neuroinflammation, causing immune cell infiltration in the sciatic nerve and a faster activation of astrocytes and microglia in the spinal cord.

**Conclusion:**

Intense endurance exercise, acting on skeletal muscles, worsens the pathological hallmarks of ALS, such as denervation and neuroinflammation, brings the onset forward, and accelerates the progression of the disease. Our findings show the potentiality of skeletal muscle as a target for both prognostic and therapeutic strategies; the preservation of skeletal muscle health by specific intervention could counteract the dying-back process and protect motor neurons from death. The physiological characteristics and accessibility of skeletal muscle further enhance its appeal as a therapeutic target.

## Graphical Abstract

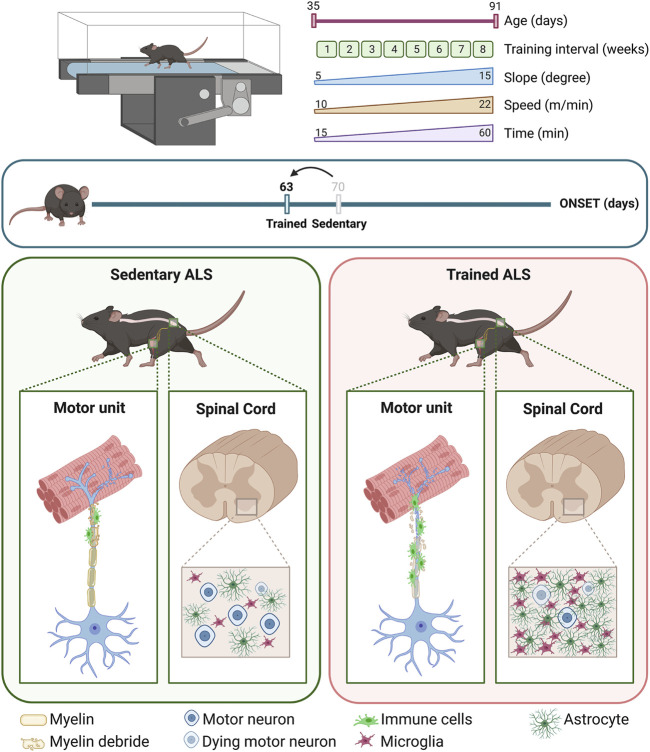

## 1 Introduction

Amyotrophic lateral sclerosis (ALS) is an incurable disease characterized by the progressive loss of upper and lower motor neurons, which is just the tip of the iceberg of a chain of different and intertwined pathological events (Mead et al., 2023). For instance, the disruption of energy homeostasis has a strong impact on disease progression (Ferri and Coccurello, 2017; Steyn et al., 2018), as shown by ALS patients and animal models that share alterations in lipid and glucose metabolism and a defective energy balance with high energy expenditure and impaired ATP production (Dupuis et al., 2011; Scaricamazza et al., 2020). Furthermore, the muscle fibers of ALS patients and animal models show poor metabolic flexibility and a propensity to use fatty acids as the main energy source (Steyn et al., 2018).

Physical activity (PA) is defined as any movement performed by skeletal muscles through energy consumption. Exercise resembles an organized scheme of PA that aims at improving or maintaining physical fitness through structured, planned, and repetitive movements (Dasso, 2019). Although exercise acts on the skeletal muscles, it indirectly influences the metabolism and physiology of the whole organism. Skeletal muscles respond to different stimuli by changing their metabolism and morphology through the differential expression of specific fiber types. For instance, different myosin heavy-chain isoforms allow specific contractility schemes (slow/fast), relying on a specific metabolic environment (Mukund, 2019). Consistently, skeletal muscle adaptations and changes in muscle-specific energy homeostasis could reflect on systemic metabolism (Mukund, 2019), thus representing a potential target for therapeutic approaches (Dupuis and Echaniz-Laguna, 2010; Scaricamazza et al., 2021).

In ALS mouse models, the metabolic features of the skeletal muscle motor units more closely resemble the characteristics of long-lasting endurance exercise, even at resting state. The ALS muscle is mainly characterized by a glycolytic-to-oxidative switch of muscle fibers and a significant shift from the expression of myosin type IIb (MyHC IIb/MYH4) (characterizing fast glycolytic fibers) to myosin type IIa (MyHC IIa/MYH2) (marking fast oxidative fibers) (Peggion et al., 2017; Scaricamazza et al., 2020). In a physio-pathological view, these metabolic modifications are not induced by an intense energy-demanding PA but are probably due to early mitochondrial dysfunctions that impair ATP production (Dupuis et al., 2004; Scaricamazza et al., 2020).

The link between PA and ALS is still an open question. So far, the epidemiological studies have led to inconsistent results, probably because of the heterogeneity of PA analyzed, the difficulties in retrospective studies, and the variability in the onset and progression of ALS. Although several research studies describe PA as a risk factor for ALS, others do not report a significant correlation, and others even highlight its potential benefits for ALS patients (Ferri et al., 2019; Chapman et al., 2023). Furthermore, studies on mice trained with different exercise protocols have given inconsistent results depending on the type, duration, and intensity of the exercise (Scaricamazza et al., 2021). Moreover, the importance of biological sex in influencing the onset and progression of the disease in response to endurance exercise was wildly highlighted in animal models of ALS, although these findings frequently present conflicting results (Kirkinezos et al., 2003; Veldink et al., 2003; Mahoney et al., 2004).

Herein, we dissect the effect of intense endurance exercise on female mice of the ALS mouse model SOD1-G93A by analyzing the *in vivo* functional readouts and the molecular alterations of the entire motor unit. To this aim, we trained mice for 8 weeks on a motorized treadmill, starting before the onset of any ALS symptoms. We demonstrate that intense endurance training accelerates the onset and progression of the disease, fostering the oxidative status of skeletal muscles, inducing muscle denervation and motor neuron loss, and increasing central and peripheral neuroinflammation.

## 2 Materials and methods

### 2.1 Animals

All animal procedures were carried out following the European guidelines for the use of animals in research (2010/63/EU) and the requirements of Italian laws (D.L. 26/2014); they were approved by the Italian Ministry of Health (protocol number 293/2021-PR).

Transgenic hemizygous SOD1-G93A male mice (B6.Cg-Tg [SOD1 ^G93A^]1Gur/J) were obtained from The Jackson Laboratory (Bar Harbor, ME, United States of America; RRID:MGI:4835776) and then crossbred with C57BL/6 female mice to gain offspring. Generated animals were genotyped by PCR through hSOD1 oligos using interleukin-2 (IL-2) as a PCR internal control (sequences in Table 1). At the end of the training protocols, trained and untrained mice were anesthetized using Rompum (xylazine, 20 mg mL^−1^, 0.5 mL kg^−1^, Bayer, Milan, Italy) in combination with Zoletil (tiletamine and zolazepam, 100 mg mL^−1^, 0.5 mL kg^−1^, Virbac, Milan, Italy). After the pedal-withdrawal reflex was no longer present, the mice were euthanized for tissue dissection.

**TABLE 1:** Target genes and primer sequences used for real-time PCR.

Accession number	Target	Forward sequence	Reverse sequence
NM_000454	Sod1	5′-CAT​CAG​CCC​TAA​TCC​ATC​TGA-3′	5′-CGC​GAC​TAA​CAA​TCA​AAG​TGA-3′
AF195955	Il-2	5′-TAG​GCC​ACA​GAA​TTG​AAA​GAT​CT-3′	5′-GTA​GGT​GGA​AAT​TCT​AGC​ATC​ATC C-3′
NM_001307989	Nor1	5′-TAC​GCC​ACG​CAG​ACT​TAT​GG-3′	5′-TGG​TCA​GCT​TGG​TGT​AGT​CG-3′
NM_025540	Sln	5′-TGA​GGT​CCT​TGG​TAG​CCT​GA-3′	5′-CAC​ACC​AAG​GCT​TGT​CTT​CA-3′
AB008453	Glut4	5′-GGT​GTG​GTC​AAT​ACG​GTC​TTC​AC-3′	5′-AGC​AGA​GCC​ACG​GTC​ATC​AAG​A-3′
NM_001039545	Myhc IIa	5′-AGT​CCC​AGG​TCA​ACA​AGC​TG-3′	5′- GCA​TGA​CCA​AAG​GTT​TCA​CA-3′
NM_010855	Myhc IIb	5′-AGT​CCC​AGG​TCA​ACA​AGC​TG-3′	5′-TTT​CTC​CTG​TCA​CCT​CTC​AAC​A-3′
NM_009930	Col3a1	5′-CCC​AAC​CCA​GAG​ATC​CCA​TT-3′	5′-GGT​CAC​CAT​TTC​TCC​CAG​GA-3′
NM_026346	Atrogin-1	5′-TGA​GCG​ACC​TCA​GCA​GTT​AC-3′	5′-GCG​CTC​CTT​CGT​ACT​TCC​TT-3′
AF325348	Achrα	5′-GGC​TTT​CAC​TCT​CCG​CTG​AT-3′	5′-TCA​GCG​GCG​TTA​TTG​GAC​TC-3′
NM_021544	Na_v_1.5	5′-AGA​TGT​CTC​CCC​CAG​TAA​CCA-3′	5′-CTT​GGG​GAG​CCT​GTC​TCT​C-3′
NM_013684	Tata box-binding protein	5′-CCA​ATG​ACT​CCT​ATG​ACC​CCT​A-3′	5′-CAG​CCA​AGA​TTC​ACG​GTA​GAT-3′

### 2.2 Exercise training protocol

Female SOD1-G93A and WT littermate mice were randomly categorized into an intense endurance training group and a sedentary control group (n = 12/group). At 35 days of age, mice started running on a motorized treadmill (Ugo Basile, Rodent Treadmill NG cat. 47300) five times a week for 8 weeks, increasing speed, uphill slope, and time as shown in Table 2; we modified the protocol from Alizadeh et al. (2015) to obtain an intense endurance exercise. The treadmill was equipped with a mild shock to encourage the animal to keep running.

**TABLE 2:** Intense endurance exercise protocol on motor treadmill modified from the work of Alizadeh et al. (2015).

Weeks of training	Parameter	Monday	Tuesday	Wednesday	Thursday	Friday
**1st week**	Speed (meters/minute)	10	10	10	10	10
	Slope (degree)	5	5	10	10	10
	Time (minutes)	15	15	15	15	15
**2nd week**	Speed (meters/minute)	10	10	12	12	12
	Slope (degree)	10	10	10	10	10
	Time (minutes)	15	15	15	30	30
**3rd week**	Speed (meters/minute)	12	12	12	12	12
	Slope (degree)	10	10	10	10	10
	Time (minutes)	30	30	30	45	45
**4th week**	Speed (meters/minute)	13	13	13	13	13
	Slope (degree)	15	15	15	15	15
	Time (minutes)	45	60	60	60	60
**5th week**	Speed (meters/minute)	17	17	17	17	17
	Slope (degree)	15	15	15	15	15
	Time (minutes)	60	60	60	60	60
**6th week**	Speed (meters/minute)	19	19	19	19	19
	Slope (degree)	15	15	15	15	15
	Time (minutes)	60	60	60	60	60
**7th week**	Speed (meters/minute)	22	22	22	22	22
	Slope (degree)	15	15	15	15	15
	Time (minutes)	60	60	60	60	60
**8th week**	Speed (meters/minute)	22	22	22	22	22
	Slope (degree)	15	15	15	15	15
	Time (minutes)	60	60	60	60	60

To reduce animal stress and experimental variability, the training was carried out in the morning between 8:00 a.m. and 1:00 p.m., and it included, on top of the training, 1 hour of acclimatization in the experimental room, 10 min of warm-up, and 10 min of cool-down.

The day after the last training section, trained and sedentary mice were anesthetized and euthanized, and the tissues were collected.

### 2.3 Disease progression evaluation

The disease progression was evaluated starting from 35 days of age (i.e., the start of the training) until the end of the training period (91 days of age) by measuring weight and grip strength and scoring the motor skills.

#### 2.3.1 Weight

Body weight was measured twice a week before the training session and other tests.

#### 2.3.2 Grip strength by the grid net

The test consisted of three attempts per mouse with a resting time of 1 minute between every attempt; the mean of the three examinations, representing the strength of four limbs, was normalized to the weight of the mouse as previously described (Scaricamazza et al., 2022). The test was carried out by the same operator to minimize experimental variability.

#### 2.3.3 Motor skills

Following Milanese et al.’s (2021) testing of the hind limb extension reflex, we assessed the hind limb posture and extension while holding the animal suspended by the tail (Milanese et al., 2021). To assess the gait, the mice were allowed to walk freely in an open field, and we assigned a score to their motor deficits by observing them (Milanese et al., 2021). The scoring scale ranged from a maximum of 5 points (no motor dysfunctions) to 0 (complete impairment), as shown in Table 3.

**TABLE 3:** Score scale to evaluate the hind limb extension reflex and gait impairment, modified from the work of Milanese et al. (2021).

Hind limb extension reflex		Gait	
Score	Description	Score	Description
**5**	Normal functions	**5**	Normal functions
**4.75**	Slight and transient limb extension deficits	**4.75**	Slight and transient stagger gait
**4.5**	Slight extension deficits and the symmetry of the hind limbs	**4.5**	Slight stagger gait at the rear torso
**4**	Slight extension deficits and slight signs of hind limb asymmetry	**4**	Moderate stagger gait at the rear torso and straight and fast gait
**3.5**	Moderate extension reflex deficits or moderate signs of hind limb asymmetry: possible slight tremors	**3.5**	Slight motor deficits of one or both hind limbs and straight gait
**3**	Moderate extension reflex deficits and/or moderate signs of hind limb asymmetry: possible moderate tremors	**3**	Moderate motor deficits of one or both hind limbs and slow straight gait
**2.5**	Severe extension reflex deficits, stretching and stiffness of one of both hind limbs, slight signs of paralysis for one of both hind limbs, possible severe tremors	**2.5**	Stiffness of one or both hind limbs, loose gait, and possible dragging tail
**2**	Slight paralysis of one or both hind limbs, contraction, or unnaturally stretching and possible slight signs of spine arching	**2**	Severe motor deficits due to slight paralysis of one or both hind limbs, gait mainly using the forelimbs, possible dragging tail, and possible signs of spine arching
**1.5**	Moderate paralysis of one or both hind limbs, contraction, or unnatural stretching and possible moderate signs spine arching	**1.5**	Severe motor deficits due to moderate paralysis of one or both hind limbs, gait only using the forelimbs, dragging tail, and possible moderate signs of spine arching
**1**	Severe paralysis of both hind limbs, contraction or unnaturally stretching, and slight hind limb movement only when touched and possible severe arching of the spine	**1**	The animal drags itself using the forelimbs only; dragging tail; and spine arching
**0.5**	Complete paralysis of both hind limbs and humane end-point score for euthanasia	**0.5**	Very limited circular movements, signs of impairment of forelimbs, and humane end-point score for euthanasia

The bold values represent the scores blindly assign for the motor skills (extension of the hindlimb and the gait). The score goes from 5 (no motor dysfunctions) to 0 (complete motor dysfunctions), modified from Milanese et al. (2021).

#### 2.3.4 Onset

The onset of ALS in SOD1-G93A mice was defined by considering weight, grip strength, and motor skills together using a score method ranging from 0 to 9 (0 = no impairments in any category; 9 = impairments in all categories). Specifically, we assigned 1 to weight when the weight of SOD1-G93A mice was 20% lower than the mean weight of WT mice. We assigned 1 to grip strength when SOD1-G93A mice had a muscular strength reduction of 20%. We assigned 1 to the extension reflex of the hind limb and 1 to the gait when SOD1-G93A mice reached a score of 4.75.

While the grip strength and the weight are two quantitative tests, the motor skill tests are qualitative tests where the score is given by the operator. Even though the evaluation is blind, to avoid biases, we decide to multiply the weight and grip strength scores by 3 and the extension reflex of the hind limb and the gait scores by 1.5; then, we define that the disease had started when, by adding up the points of every category, the mouse reached a total score of 6. Finally, we represented the onset with a Kaplan–Meier graph.

### 2.4 RNA isolation and real-time qPCR

Total RNA was obtained from the tibialis anterior (TA) of 91-day-old trained and sedentary SOD1-G93A and WT mice (n ≥ 5 for each experimental group) with TRIzol^®^ Reagent (Ambion, Life Technologies) and reverse-transcribed using the ImProm-II™ Reverse Transcription System (Promega). Real-time qPCR was used to evaluate the levels of mRNA expression with a LightCycler 480 SYBR Green System (Roche, ETC). We used the “second derivative max” algorithm of the LightCycler software to calculate the crossing point (Cp) values. We chose the TATA box-binding protein as the housekeeping gene for normalization. Primer sequences are listed in Table 1.

### 2.5 Electrophoresis and Western blotting

Protein samples, obtained from the TA of sedentary and trained 91-day-old SOD1-G93A and WT littermate mice (n ≥ 5 for each experimental group), were separated by SDS–PAGE and transferred onto nitrocellulose membranes (PerkinElmer, Cat# NBA085B). The membranes were blocked with 5% milk, incubated with the appropriate primary antibodies (Table 4), incubated with the appropriate peroxidase-conjugated secondary antibodies (Table 4), and developed using enhanced chemiluminescence (Bio-Rad Clarity^TM^ Western ECL Substrate, Cat# 170–5061. Densitometric analyses were performed using ImageJ software (U.S. National Institutes of Health, Bethesda, Maryland, United States of America, https://imagej.nih.gov/ij/), and we analyzed five samples per group, divided into two different blots. The expression of proteins of interest was normalized to GAPDH expression levels.

**TABLE 4:** Primary and secondary antibodies used for Western blot and IF assays.

	Antibody	Product	Dilution
Primary	Myosin heavy chain, type 2B BF-F3	DSHB	IF 1:50
	Myosin heavy chain, type 2A 2F7	DSHB	IF 1:10
	Laminin	Sigma-Aldrich (Cat#L9393)	IF 1:500
	ATPB	Abcam (Cat#ab14730)	IF 1:500
	Synaptophysin	Thermo Fisher Scientific (Cat#MA1-34660)	IF 1:200
	NFH	BioLegend (Cat#836001)	IF 1:1,000
	MBP	Cell Signaling Technology (Cat#78896)	IF 1:200
	CD68	Bio-Rad/AbD Serotec (Cat#MCA1957GA)	IF 1:200
	GFAP	Novus Biological (Cat#NBP1-05197)	IF 1:500
	LRP4	BioLegend (Cat#832201)	WB 1:1,000
	Dystrophin	Proteintech (Cat#12715-1-AP)	WB 1:2000
	GAPDH	Santa Cruz Biotechnology (Cat#sc-20356)	WB 1:10,000
Secondary	Goat anti-mouse IgM (heavy chain) cross-adsorbed secondary antibody, Alexa Fluor™ 555	Thermo Fisher (Cat#A-21426)	IF 1:400
	Goat anti-mouse IgG1 cross-adsorbed secondary antibody, Alexa Fluor™ 488	Thermo Fisher (Cat#A-21121)	IF 1:400
	Alexa Fluor^®^ 488 AffiniPure Donkey Anti-Rabbit IgG (H + L)	Jackson ImmunoResearch Laboratories (Cat#711-545-152)	IF 1:200
	Cy™3 AffiniPure F(ab')₂ Fragment Donkey Anti-Mouse IgG (H + L)	Jackson ImmunoResearch Laboratories (Cat#715-166-150)	IF 1:200
	Α-Bungarotoxin Alexa Fluor 594 Conjugate	Invitrogen™ (Cat#B13423)	IF 1:200
	Cy™5 AffiniPure F(ab')₂ Fragment Donkey Anti-Rabbit IgG (H + L)	Jackson ImmunoResearch Laboratories (Cat#711-175-152	IF 1:200
	Cy™3 AffiniPure F(ab')₂ Fragment Donkey Anti-Mouse IgG (H + L)	Jackson ImmunoResearch Laboratories (Cat#715-166-150)	IF 1:200
	Alexa Fluor^®^ 488 AffiniPure Donkey Anti-Rat IgG (H + L)	Thermo Fisher Scientific (Cat#A-21208)	IF 1:200
	Goat Anti-Rabbit IgG (H + L)-HRP Conjugate	Bio-Rad Laboratories (Cat#1706515)	WB 1:2500
	Goat Anti-Mouse IgG (H + L)-HRP Conjugate	Bio-Rad Laboratories (Cat#170-6516)	WB 1:2500

WB: Western blot.

### 2.6 Skeletal muscle immunofluorescence and histochemistry

The TA of 91-day-old trained and sedentary SOD1-G93A and WT mice (n ≥ 5 for each experimental group) was embedded into the Tissue-Tek Optimal Cutting Temperature (O.C.T.) Compound and sectioned at 10 μm using a Leica Cryostat.

For nicotinamide adenine dinucleotide dehydrogenase (NADH)-tetrazolium reductase activity staining, TA cryosections were hydrated in 0.1 M Tris-HCl, pH 7.5, for 10 min and incubated in NADH-tetrazolium reductase activity solution (0.4 mg/mL NADH, 0.8 mg/mL NTB, and 1 M Tris-HCl, pH 7.5) for 40 min at 37°C in a humidified chamber. The slides were dehydrated in increasing concentrations of ethanol, cleared in xylene, and mounted in a Eukitt mounting medium. Images of the whole muscle section were acquired using an Olympus BX51 Microscope. The percentage of NADH-positive areas was measured using ImageJ software (U.S. National Institutes of Health, Bethesda, Maryland, United States of America, https://imagej.nih.gov/ij/).

For sirius red collagen staining, TA sections were fixed 1 h with Bouin solution at 56°C and stained for 1 h with picrosirius red 0.1% (0.1% Direct Red in picric acid), dehydrated in increasing concentrations of ethanol, cleared in xylene, and mounted in a Eukitt mounting medium. Images of the whole muscle section were acquired using an Olympus BX51 Microscope. The percentage of the red area was measured using Fiji software.

For immunofluorescence (IF) staining, TA sections were blocked in PBS containing 0.3% Triton X-100 and 10% NDS for 1 h at room temperature (RT) and incubated overnight at 4°C with the primary antibody (Table 4) in PBS containing 0.3% Triton X-100 and 2% NDS, followed by incubation with the appropriate secondary antibody for 3 h at room temperature in the same solution. Immunofluorescence on the TA was visualized at 10X or ×20 magnification using a Zeiss LSM 800 Confocal Laser Scanning Microscope and processed by ZEN 2.6 (blue edition) (Carl Zeiss, Milan, Italy), taking at least four photos per sample.

The cross-sectional area (CSA) was analyzed using ImageJ Plugin “Macro_seg_5_modif.ijm.txt.” To evaluate the percentage of positive fibers for MyHC IIa and MyHC IIb, we used Plugin “Weka_Segmentation.”

The co-localization area of α-bungarotoxin and synaptophysin in NMJs was analyzed using ImageJ (U.S. National Institutes of Health, Bethesda, Maryland, United States of America, https://imagej.nih.gov/ij/).

We used ATPB staining to analyze the mitochondria. The area of the mitochondria in the entire TA was measured using Fiji, while the footprint of mitochondria in the single fibers was processed using Mitochondrial Network Analysis (MiNA), an ImageJ plugin, following the toolset in the work of Valente et al. (2017). We chose the fibers with a diameter <1000 μm (at least three fibers per image); then, the analysis consisted of steps that preserved the quality image, converted it into a binary image, and created a mitochondrial network for the quantitative analysis.

### 2.7 Spinal cord and sciatic nerve immunofluorescence and histochemistry

The spinal cords and sciatic nerves of sedentary and trained 91-day-old SOD1-G93A and WT mice (n ≥ 5 for each experimental group) were post-fixed in 4% paraformaldehyde, cryoprotected in 30% sucrose in PB, embedded in the O.C.T. compound, and cut using a cryostat at 30 μm thickness for the spinal cord (L3–L5) and 15 μm thickness for the sciatic nerve.

To count the motor neurons, we performed cresyl violet staining (Nissl staining). In brief, the sections were stained with 1% cresyl violet, dehydrated in 50%–100% alcohol, cleared in xylene, and mounted in a Eukitt mounting medium (Sigma-Aldrich). The images of the ventral horns were obtained at ×10 magnification using a Zeiss Axioskop 2 Microscope. The neurons with a cell body area ≥200 μm^2^ and a definable cytoplasm with a nucleus and nucleolus were counted using Neurolucida software (MBF Bioscience, United States of America). For immunofluorescence on the spinal cord and sciatic nerve, the sections were blocked for 1 h at RT in PBS containing 0.3% Triton X-100 and 10% NDS and incubated for 48 h at 4°C with the primary antibody (listed in Table 4) in PBS containing 0.3% Triton X-100 and 2% NDS, followed by incubation with the appropriate secondary antibody (listed in Table 4) for 3 h at RT in the same solution, as previously described (Scaricamazza et al., 2022).

Immunofluorescence on the spinal cord and sciatic nerve sections was visualized at ×20 magnification using a Zeiss LSM 800 Confocal Laser Scanning Microscope, processed using ZEN 2.6 (blue edition) (Carl Zeiss, Milan, Italy), and analyzed using ImageJ (U. S. National Institutes of Health, Bethesda, Maryland, United States of America, https://imagej.nih.gov/ij/, 1997–2016).

### 2.8 Data analysis

Data are presented as the mean ± SEM. For the evaluation of the onset, data were analyzed using the Kaplan–Meier curve with a log-rank (Mantel–Cox) test and a log-rank test for trend. Then, the non-parametric Mann–Whitney test was used to evaluate the difference in the onset between groups (SD G93A and TR G93A). For the analysis of weight, grip strength tests, motor skill tests, histology, and protein and RNA expression, data were evaluated using a two-way ANOVA with the Tukey test as a *post hoc* analysis. Differences between groups were considered significant when the *p*-value was less than 0.05. The normality of the data was assessed using the Shapiro–Wilk test. Statistical analysis was carried out using GraphPad Prism 5 software.

## 3 Results

### 3.1 Intense endurance exercise brings ALS onset forward

SOD1-G93A and wild-type (WT) female mice underwent an intense endurance exercise protocol on a rodent treadmill for 8 weeks, 5 days a week, starting at 35 days of age, which is well before any ALS symptoms (Scaricamazza et al., 2022). To understand the effect of intense endurance exercise on ALS, we analyzed several parameters indicating functional and structural hallmarks of the disease throughout the training. Specifically, we monitored weight, muscle strength, and motor skills and compared them with the sedentary groups.

**FIGURE 1:**
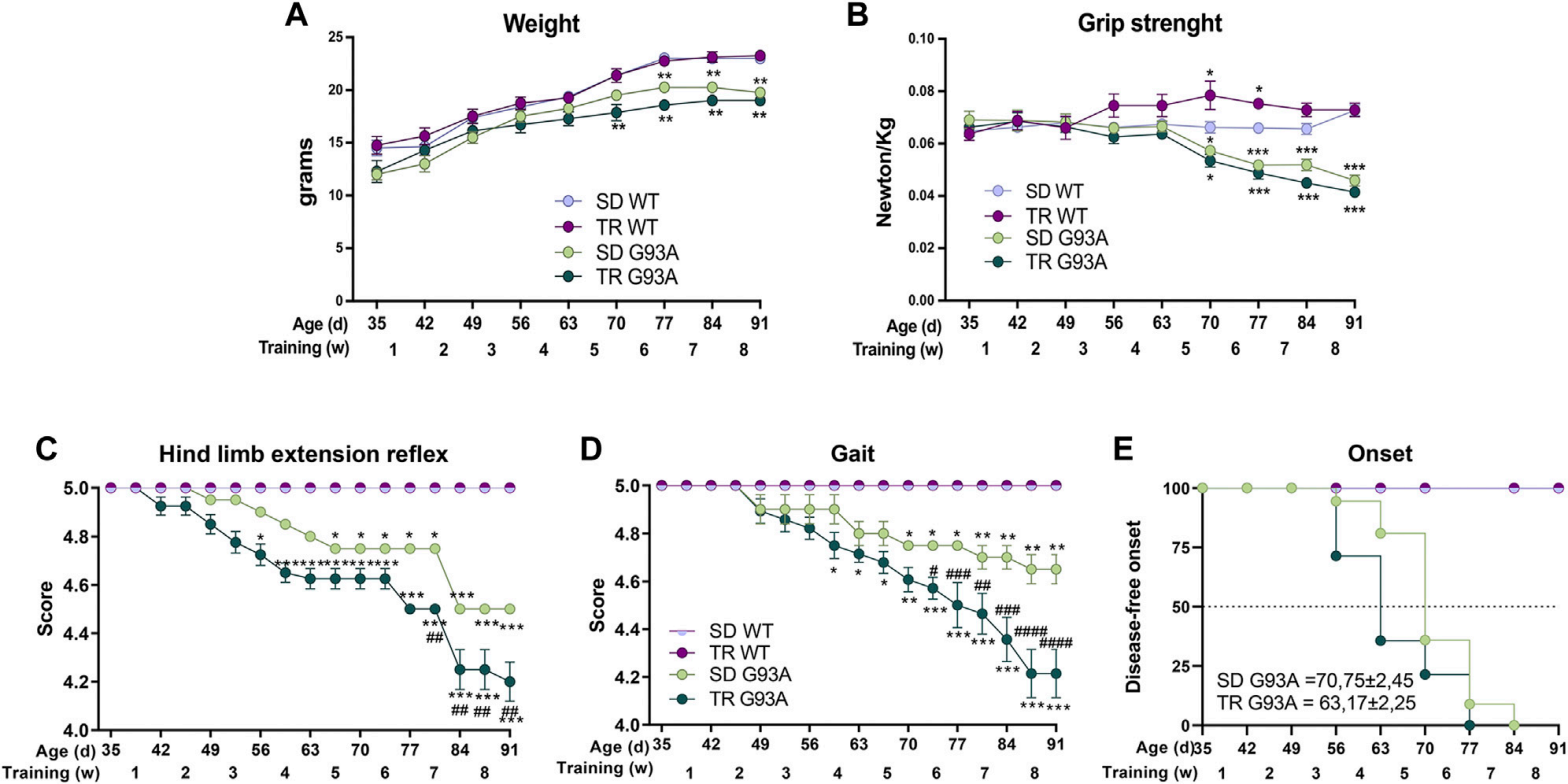
Intense endurance exercise accelerates the onset of the disease of SOD1-G93A mice. **(A)** Graph representing the weight of sedentary and trained SOD1-G93A mice (SD G93A and TR G93A) and their control littermates (SD WT and TR WT) at the indicated ages and indicated week of training. Data are presented as the means ± SEM; *p < 0.05 and **p < 0.01 when compared with SD WT. **(B)** Maximal grip strength of sedentary (SD G93A) and trained (TR G93A) SOD1-G93A mice and their control littermates (SD WT and TR WT) at the indicated ages during the 8 weeks of training. Data are presented as the means ± SEM; **p < 0.01 when compared with SD WT. **(C)** Graph representing the evaluation of hind limb extension reflex of trained (TR WT and TR G93A) and sedentary mice (SD WT and SD G93A) at the indicated ages during the training period. Mice were suspended by the tail, and the hind limb posture was evaluated giving scores from 5 (no sign of disease) to 0 (complete impairment). Data are presented as the means ± SEM; *p < 0.05 and ***p < 0.001 when compared with SD WT. ^##^ *p* < 0.01 when compared with SD G93A. **(D)** Graph representing the evaluation of gait of trained (TR WT and TR G93A) and sedentary mice (SD WT and SD G93A) at the indicated ages during the training period. Mice movement in an open field was evaluated with a scale from 5 (no sign of disease) to 0 (complete impairment). Data are presented as the means ± SEM; *p < 0.05 and **p < 0.01 when compared with SD WT. ^#^ *p* < 0.05, ^##^ *p* < 0.01, ^###^ *p* < 0.001, and ^####^ *p* < 0.0001 when compared with SD G93A. *p*-values in A, B, C, and D were obtained using parametric two-way ANOVA with a Tukey *post hoc* test. n = 12 per group **(E)** Kaplan–Meier graph representing the onset of the disease of the sedentary and trained SOD1-G93A mice (SD G93A and TR G93A) and their control littermates (SD WT and TR WT). ^#^ *p* < 0.05 compared with SD G93A. *p*-value was obtained by analyzing the mean of the onset of SD G93A and TR G93A with the non-parametric Mann–Whitney test.

As shown in Figure 1A, unlike the WT mice that gradually gained weight, the weight of SOD1-G93A mice increased only in the first training weeks. Interestingly, trained SOD1-G93A mice stopped gaining weight earlier than the sedentary mice, at 70 and 77 days of age, respectively (Figure 1A).

The training improved the muscular strength of WT mice but did not affect the strength of SOD1-G93A mice (Figure 1B). We noticed that the trained transgenic mice tended to be weaker than the sedentary group, even though the difference was not statistically significant (Figure 1B).

The training significantly worsened the motor abilities of SOD1-G93A mice. Following the protocol of Milanese et al. (2021), we used two different tests to evaluate the motor skills: the hind limb extension reflex and the gait. As shown in Figure 1C, the score of the hind limb extension reflex of trained SOD1-G93A mice started decreasing at 52 days of age, 2 weeks before the sedentary transgenic mice. The gait test showed a similar pattern, with the trained transgenic mice starting to lower their score at 59 days of age and the sedentary transgenic mice at 70 days of age (Figure 1D).

By taking together the weight, grip strength, and score of the motor skill tests, we defined the onset of the disease (see Section 2 for details). As shown in Figure 1E, the training accelerated the onset of the disease by approximately 1 week, starting in trained mice at 63 days of age and sedentary mice at 70 days of age.

### 3.2 Intense endurance exercise exacerbates the oxidative shift of muscle fibers

Skeletal muscle is a plastic tissue able to change and model its fibers in response to functional and metabolic needs. Intense endurance exercise pushes muscle fibers toward a more oxidative phenotype, promoting the shift from glycolytic to oxidative fibers (Ausoni et al., 1990; Schiaffino and Reggiani, 2011; Mukund, 2019). We have recently demonstrated that the same shift occurs early in the glycolytic skeletal muscles of sedentary SOD1-G93A mice before any denervation events (Scaricamazza et al., 2020). Thus, we wondered if the oxidative status that the training induced caused the early deficit of motor skills in trained SOD1-G93A mice by hastening the damage to skeletal muscles. In detail, we chose to analyze the *tibialis anterior* because it is a glycolytic muscle that we previously characterized and demonstrated to be affected in our model (Scaricamazza et al., 2020; 2022).

**FIGURE 2:**
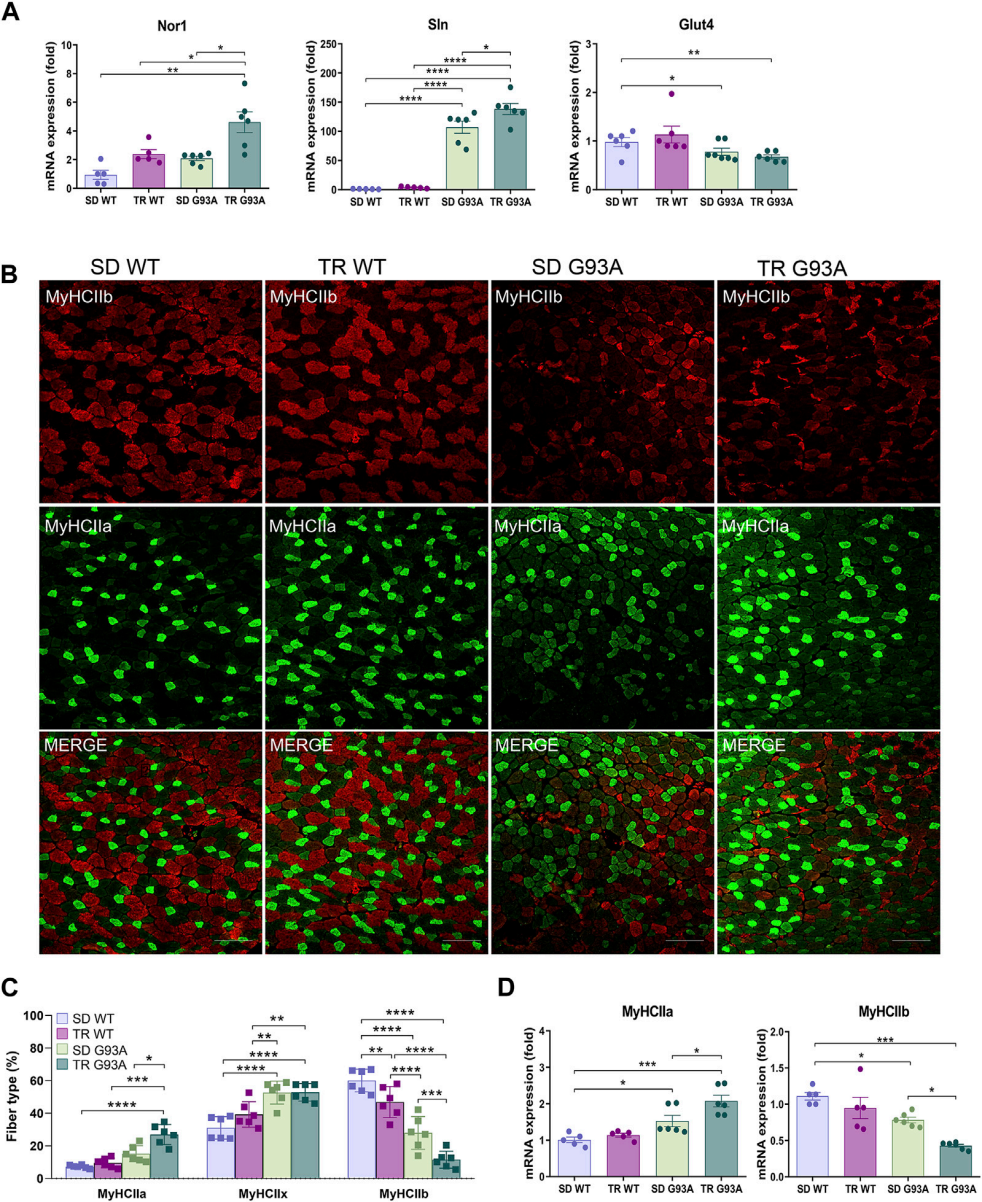
Intense endurance exercise intensifies the TA oxidative phenotype of SOD1-G93A mice. **(A)** Expression level of mRNA encoding Nor1, Sln, and Glut4 in the TA muscle of 91-day-old sedentary and trained wild-type and SOD1-G93A mice (SD WT, TR WT, SD G93A, and TR G93A). Data are presented as the means ± SEM. *p < 0.05, **p < 0.01, and ****p < 0.0001. **(B)** TA cross sections of 91-day-old sedentary and trained wild-type and SOD1-G93A mice (SD WT, TR WT, SD G93A, and TR G93A) stained for MyHC IIb and MyHC IIa. Scale bar, 200 μm. **(C)** Graphical representation of the percentage of MyHC IIb, MyHC IIa, and MyHC IIx area. MyHC IIx area was assessed measuring the uncolored fibers. Data are presented as the means ± SEM. *p < 0.05, **p < 0.01, ***p < 0.001, and ****p < 00,001. **(D)** Expression level of mRNA encoding MyHC IIa and MyHC IIb in the TA muscle of 91-day-old sedentary and trained wild-type and SOD1-G93A mice (SD WT, TR WT, SD G93A, and TR G93A). Data are presented as the means ± SEM. *p < 0.05; ***p < 0.001. *p*-values were obtained using parametric two-way ANOVA with a Tukey *post hoc* test. n ≥ 5 per group.

To define the oxidative status of the muscle fibers, we performed, at the end of the eighth-week training protocol, an *in situ* NADH-tetrazolium reductase activity assay on the TA muscle of sedentary and trained WT and SOD1-G93A mice (91 days of age). Since the NADH-positive area in the TA of the sedentary SOD1-G93A mice covered almost the entire sample, we could not see any difference between trained and sedentary mice (Supplementary Figures S1A, B). However, we observed an upregulation in the expression levels of sarcolipin (*Sln*) and neuron-derived orphan nuclear receptor (*Nor1*) and a slight downregulation, although not statistically significant, in the expression of glucose transporter type 4 (*Glut4*) in trained SOD1-G93A mice (Figure 2A), indicating that training shifted muscle fibers toward oxidative metabolism.

Consistent with that, immunofluorescence analysis revealed that training led to a significant increase in MyHC IIa/MYH2-positive fibers (Figures 2B, C) and a strong decrease in MyHC IIb/MYH4-positive fibers in SOD1-G93A mice (Figures 2B, C), while it did not affect the MyHC IIx/MYH1-positive area, which was assessed by considering the unstained fibers (Figures 2B, C). According to the literature, MyHC IIb-positive fibers decrease in WT mice after training (Figures 2B, C) (Yan et al., 2011; Qaisar et al., 2016). In agreement, we observed the same trend when analyzing the mRNA expression of *Myh2* and *Myh4* in the TA of the same experimental groups (Figure 2D).

**FIGURE 3:**
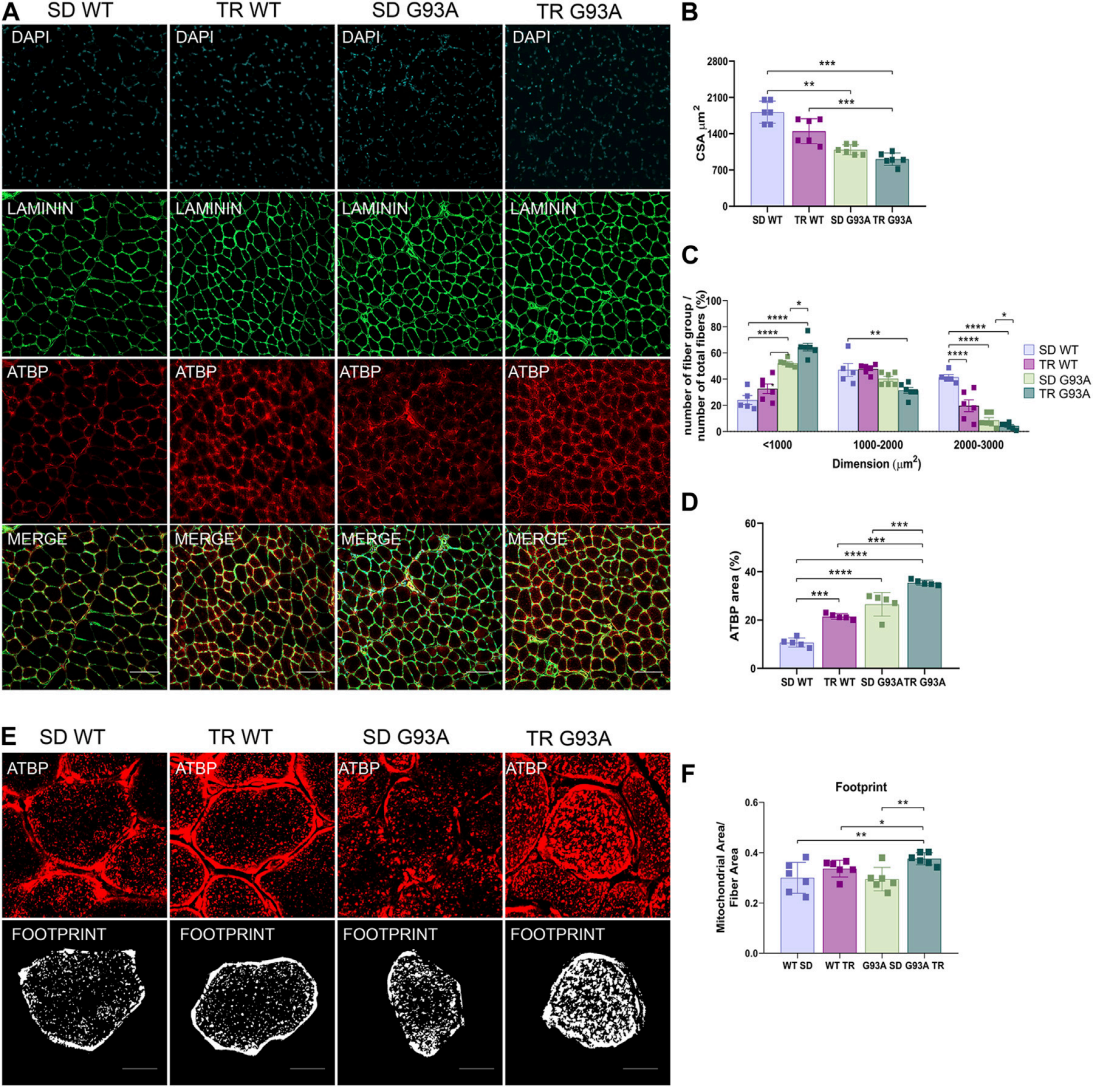
Intense endurance exercise increases mitochondria area in the TA oxidative fibers of SOD1-G93A mice. **(A)** TA cross sections of 91-day-old sedentary and trained wild-type and SOD1-G93A mice (SD WT, TR WT, SD G93A, and TR G93A) stained for laminin and mitochondrial marker ATPB. Scale bar, 200 μm. **(B)** Graphical representation of the total cross-sectional area of 91-day-old sedentary and trained wild-type and SOD1-G93A mice (SD WT, TR WT, SD G93A, and TR G93A). Data are presented as the means ± SEM. **p < 0.01; ***p < 0.001. **(C)** Graphical representation of the cross-sectional area of 91-day-old sedentary and trained wild-type and SOD1-G93A mice (SD WT, TR WT, SD G93A, and TR G93A) divided into groups of sizes ranging from <1,000, 1,000–2000, and 2000–3,000 μm^2^. The percentage of the single group of fibers was obtained giving 100% to the total number of fibers. Data are presented as the means ± SEM. *p < 0.05, **p < 0.01, ***p < 0.001, and ****p < 0.0001. **(D)** Graphical representation of ATPB area on the TA of 91-day-old sedentary and trained wild-type and SOD1-G93A mice (SD WT, TR WT, SD G93A, and TR G93A). Data are presented as the means ± SEM. ***p < 0.001; ****p < 0.0001. **(E)** ATPB staining to detect mitochondria footprint on TA oxidative fibers of 91-day-old sedentary and trained wild-type and SOD1-G93A mice (SD WT, TR WT, SD G93A, and TR G93A). Scale bar, 25 μm^2^. Images were obtained with z-stack projection (63x) and next processed using the MiNA toolset. **(F)** Graphical representation of mitochondria footprint obtained in **(E)** and expressed as mitochondrial area μm^2^. Data are presented as the means ± SEM. **p < 0.01. *p*-values were obtained using parametric two-way ANOVA with a Tukey *post hoc* test. n ≥ 5 per group.

The CSA measure corroborated the fiber shift: although the total CSA did not change (Figures 3A, B), by dividing for a specific dimension range and giving 100% to the total number of fibers, we observed that the percentage of small oxidative fibers (<1,000 μm^2^) increased, while the percentage of large glycolytic fibers (2000–3,000 μm^2^) decreased if compared to sedentary SOD1-G93A mice (Figure 3C). In agreement with these results, immunofluorescence on the same muscles revealed that training increased the mitochondrial marker ATPB, both in WT and SOD1-G93A mice (Figures 3A, D). Moreover, we detected with the MiNA 2.0 toolset that the oxidative fibers of trained SOD1-G93A mice had larger mitochondrial areas than sedentary WT and SOD1-G93A mice (Figures 3E, F), further confirming oxidative metabolism.

Overall, these data suggest that intense endurance training exacerbates the oxidative shift occurring in the glycolytic muscles of SOD1-G93A mice.

### 3.3 Intense endurance training speeds up the denervation process in SOD1-G93A mice

Considering that the skeletal muscle of SOD1-G93A mice had a worse oxidative status after training, we next assessed whether intense endurance exercise also affected muscle integrity, particularly fibrosis and denervation.

Picrosirius red staining (Supplementary Figures S2A, B) and collagen type III alpha 1 chain (*Col3a1*) mRNA expression analysis (Supplementary Figure S2C) on the TA of 91-day-old mice showed that training did not affect fibrosis as the percentage of red staining and Col3a1 mRNA expression did not change in sedentary and trained transgenic mice (Supplementary Figure S2B).

**FIGURE 4:**
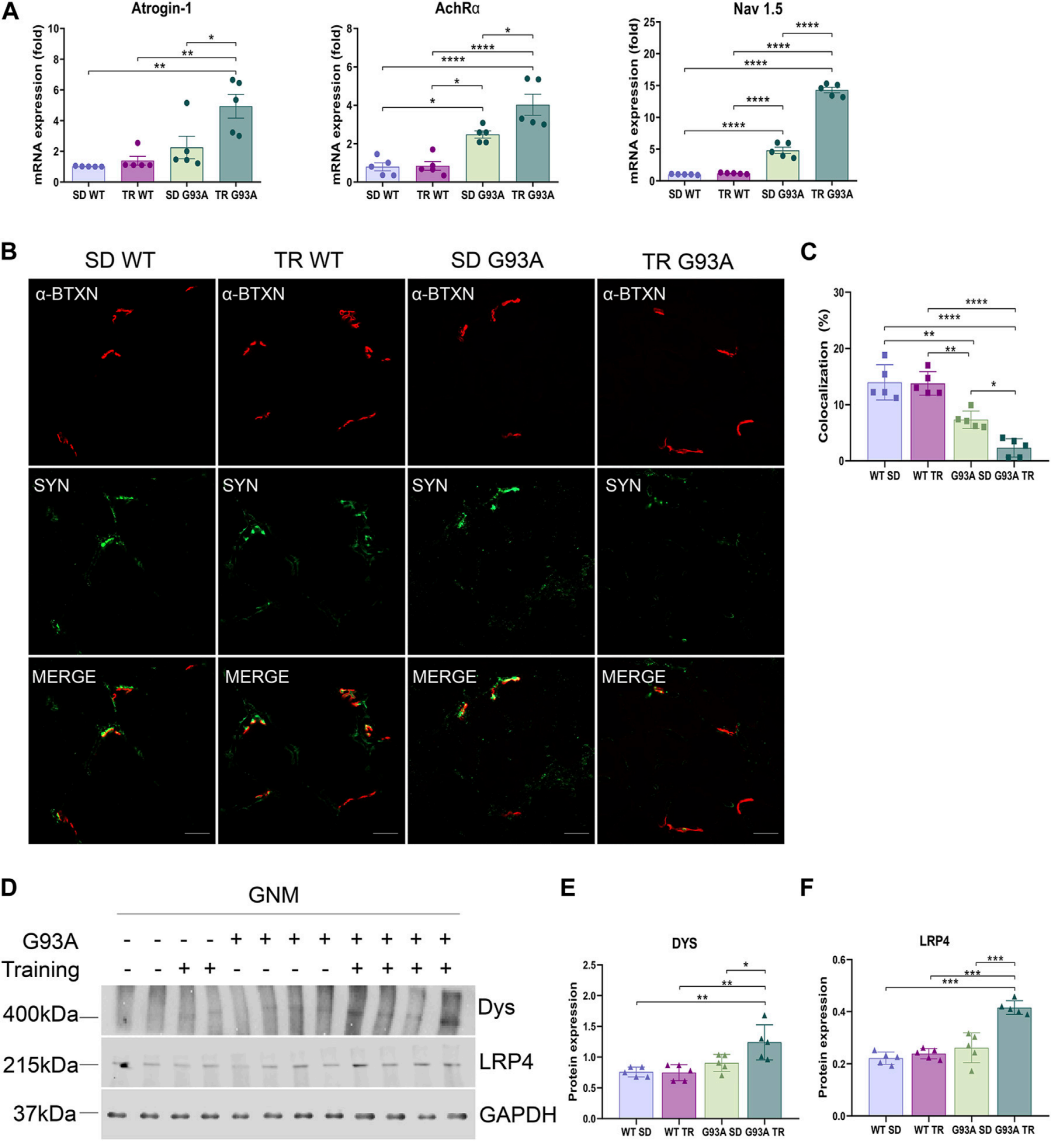
Intense endurance exercise promotes denervation in skeletal muscle of SOD1-G93A mice. **(A)** Expression level of mRNA encoding atrogin-1, AchRα, and Na_V_1.5 on the TA of 91-day-old sedentary and trained wild-type and SOD1-G93A mice (SD WT, TR WT, SD G93A, and TR G93A). Data are presented as the means ± SEM. *p < 0.05, **p < 0.01, and ****p < 0.0001. **(B)** TA cross sections of 91-day-old sedentary and trained wild-type and SOD1-G93A mice (SD WT, TR WT, SD G93A, and TR G93A) staining for α-bungarotoxin (α-BTXN) and synaptophysin (SYN) to detect NMJ (scare bar: 20 μm). **(C)** Graphical representation of α-BTXN and SYN co-localization percentage. Data are presented as the means ± SEM. *p < 0.05, **p < 0.01, and ****p < 0.0001. Representative Western blots **(D)** and quantification of **(E)** dystrophin (Dys) and **(F)** lipoprotein receptor-related protein 4 (LRP4) on TA of 91-day-old sedentary and trained wild-type and SOD1-G93A mice (SD WT, TR WT, SD G93A, and TR G93A). Data are presented as the means ± SEM. **p < 0.01; ***p < 0.001. *p*-values were obtained using parametric two-way ANOVA with a Tukey *post hoc* test. n ≥ 5 per group.

On the contrary, denervation strongly advanced in trained SOD1-G93A mice. Specifically, the expression levels of atrophy and denervation markers, such as F-box-only protein 32**/**atrogin-1, acetylcholine receptor α subunit (AChRα), and voltage-gated sodium channel 1.5 (Na_v_1.5), increased in SOD1-G93A mice after training compared to the sedentary mice (Figure 4A). To further investigate the denervation process, we evaluated the neuromuscular junction (NMJ) dismantling in the TA of trained transgenic mice. Comparing trained and sedentary transgenic mice, immunofluorescence analysis showed a reduced co-localization of nicotinic acetylcholine receptor (nAChR) clusters (defined with α-bungarotoxin, α-BTXN) and synaptophysin (SYN) in trained mice, indicating increased NMJ instability (Figures 4B, C). Dystrophin (Dys) and low-density lipoprotein receptor-related protein 4 (LRP4) are essential for NMJ stability and postsynaptic differentiation, and, as we previously reported, they increase in SOD1-G93A mice starting at 120 days of age (Scaricamazza et al., 2022), probably to counter the damage of denervation. As shown in Figures 4D–F, training accelerated the upregulation of LRP4 and Dys in SOD1-G93A mice, further confirming the reduced NMJ stability.

### 3.4 Intense endurance exercise enhanced Wallerian degeneration in the peripheral nervous system of SOD1-G93A mice

**FIGURE 5:**
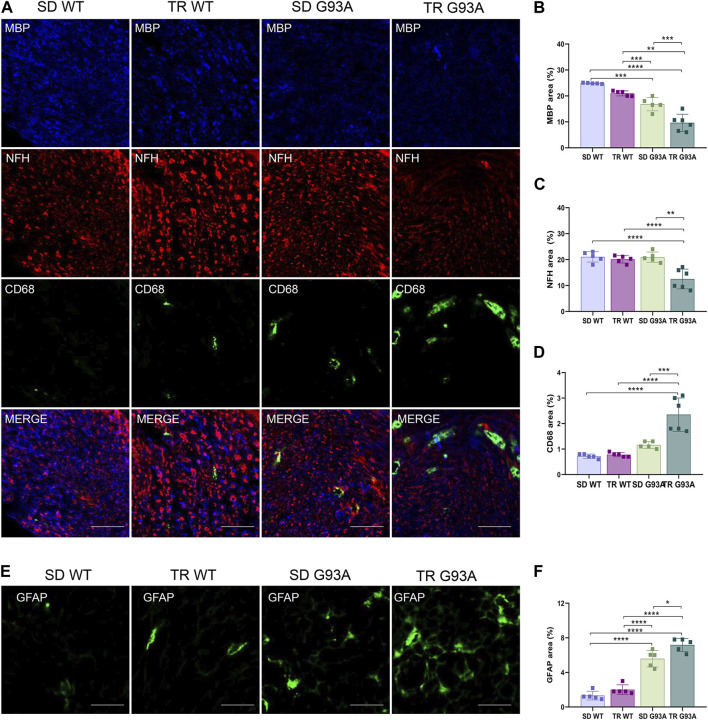
Intense endurance exercise accelerates Wallerian degeneration in the sciatic nerve of SOD1-G93A mice. **(A)** Sciatic nerve cross sections of 91-day-old sedentary and trained wild-type and SOD1-G93A mice (SD WT, TR WT, SD G93A, and TR G93A) staining for myelin (MBP), neurofilament heavy (NFH), and activated immune cells (CD68). Scale bar, 50 μm. Graphical representation of MBP **(B)**, NFH **(C),** and CD68 **(D)** expression. Data are presented as the means ± SEM. ***p < 0.001. **(E)** Sciatic nerve cross sections of 91-day-old sedentary and trained wild-type and SOD1-G93A mice (SD WT, TR WT, SD G93A, and TR G93A) staining for phagocytic GFAP + Schwan cells (GFAP) and graphical representation **(F)**. Scale bar, 50 μm. Data are presented as the means ± SEM. *p < 0.05; ****p < 0.0001. *p*-values were obtained using parametric two-way ANOVA with a Tukey *post hoc* test. n ≥ 5 per group.

We assessed whether intense endurance exercise had an impact on demyelination, axonal loss, and inflammation in the peripheral nervous system. To this end, cross sections of the sciatic nerves of 91-day-old sedentary and trained WT and SOD1-G93A mice were probed with antibodies recognizing myelin basic protein (MBP), neurofilament heavy chain (NFH), and pro-inflammatory immune cells (CD68). As shown in Figure 5, training enhanced Wallerian degeneration in SOD1-G93A mice by prompting myelin degradation and loss of the axonal cytoskeleton, as indicated by the reduction in MBP and NFH expression levels, respectively (Figures 5A–C). The damage in the sciatic nerve came with immune cell infiltration, as the stronger staining of CD68 in the sections of trained SOD1-G93A mice revealed in Figures 5A, D.

Further confirming demyelination, sciatic nerve cross sections of trained transgenic mice showed increased staining of glial fibrillary acidic protein (GFAP) (Figures 5E, F), usually expressed in non-myelinating Schwann cells (Yang and Wang, 2015).

### 3.5 Intense endurance training accelerates motor neuron loss and neuroinflammation in SOD1-G93A mice

**FIGURE 6:**
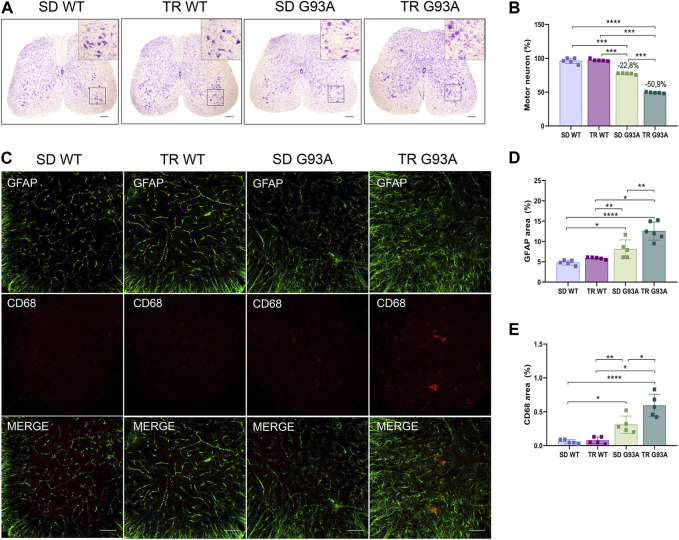
Intense endurance exercise worsens motor neuron loss and neuroinflammation in the spinal cord of SOD1-G93A mice. **(A)** Representative lumbar spinal cord sections (L3–L5) stained with cresyl violet (scale bar, 100 μm) and **(B)** graphical quantification of the motor neuron number percentage of 91-day-old sedentary and trained wild-type and SOD1-G93A mice (SD WT, TR WT, SD G93A, and TR G93A). Data are presented as the means ± SEM. ***p < 0.001; ****p < 0.0001. **(C)** Representative immunofluorescence images (scale bar, 100 μm); graphical representation of **(D)** activated astrocytes (GFAP) and **(E)** activated microglia (CD68) in the lumbar spinal cord sections (L3–L5) of 91-day-old sedentary and trained wild-type and SOD1-G93A mice (SD WT, TR WT, SD G93A, and TR G93A). Data are presented as the means ± SEM. **p < 0.01; ****p < 0.0001. *p* values were obtained using parametric two-way ANOVA with a Tukey *post hoc* test. n ≥ 5 per group.

Motor neuron loss is the final stage and the most characteristic hallmark of ALS. To determine if intense endurance exercise affected motor neurons, we performed Nissl staining on cross sections of the lumbar spinal cord (L3–L5) of sedentary and trained WT and SOD1-G93A mice. As shown in Figure 6, 91-day-old sedentary SOD1-G93A mice had a reduction of approximately 20% in the percentage of ventral horn motor neurons compared with the sedentary WT mice (Figures 6A, B). Interestingly, after training, SOD1-G93A mice reached a 50% reduction (Figures 6A, B).

In ALS, the death of motor neurons comes with a neuroinflammatory response characterized by the activation of reactive microglia and astrocytes (Volonté et al., 2019). Here, the identification of microglial and astrocytic markers by immunofluorescence (CD68 and GFAP, respectively) showed that these two cell populations were more active in trained SOD1-G93A mice than in sedentary controls (Figures 6C–E).

## 4 Discussion

The benefits of PA on fitness and mental health have been widely described. Indeed, by acting on specific mechanisms, PA can influence the physiology of several chronic conditions. Skeletal muscle metabolism and regeneration, neurogenesis, mitochondrial biogenesis, and antioxidant defense are only some of the pathways that PA improves, and interestingly, they are all involved in ALS pathology (Scaricamazza et al., 2021). The relationship between PA and ALS is still under debate (Scaricamazza et al., 2021; Chapman et al., 2023), as clinical and preclinical studies have so far led to contradictory findings, probably because of the heterogeneity of ALS (sporadic and familial) and the different types (i.e., running; swimming, and cycling) and intensities (high, moderate, or low) of PA analyzed (Tsitkanou et al., 2019; Scaricamazza et al., 2021). Moreover, most studies did not distinguish between aerobic and anaerobic activities, a shortcoming that is likely responsible for the different conclusions (Chapman et al., 2023).

Regardless of the type, only the intensity of the exercise seems to affect the onset and progression of ALS (Scaricamazza et al., 2021). Indeed, high-intensity PA carried out during leisure time correlates with an early onset of the disease in ALS patients (Huisman et al., 2013). This evidence is corroborated by a cross-sectional study that reported a significant increase in patients diagnosed with early ALS among those who had a history of vigorous leisure PA during early adulthood (Raymond et al., 2021); finally, strenuous PA seems to increase the penetrance of ALS in patients with a risk genotype, predisposing particularly those with C9ORF72 expansion to present a higher risk for PA-aggravated disease (Julian et al., 2021).

We designed our study (an endurance training inducing oxidative metabolism in SOD1-G93A female mice) to be comparable to high-intensity PA performed during leisure time far from the onset of the disease. We highlighted how endurance training significantly accelerated the onset, worsening the progression of the disease in SOD1-G93A mice.

Accordingly, Golini et al. (2023) recently reported that repeated voluntary running on home-cage running-wheel systems negatively affects disease progression by anticipating disease onset in SOD1-G93A low-copy male mice. Partially in conflict, Mahoney and others did not observe any impact of endurance training on the disease onset of SOD1-G93A mice. However, the training protocol and method used to detect the onset are different compared to ours. Nevertheless, extending their study until the death of the animals, Mahoney et al. (2004) demonstrated that intense endurance exercise worsened motor performance after the clinical onset and hastened death in male mice without any significant effects in female mice. Here, we examined female mice, and although it is widely described that this biological sex has better outcomes compared to male mice in terms of pathology (Heiman-Patterson et al., 2005; Cacabelos et al., 2016), our training paradigm was able to accelerate the symptoms, exacerbating the hallmarks of the disease.

In our study, we pinpointed the onset of the disease by examining both neuromotor functions and functional skills. In particular, the analysis of the motor skills indicated that the decline in performance was faster in trained SOD1-G93A mice than in transgenic sedentary mice. Noteworthy, the performance worsened between the third and fourth weeks of training, when the exercise protocol had not yet reached the maximum levels of duration, speed, and slope. Unexpectedly, we did not observe differences in the decline of muscle strength between trained and sedentary SOD1-G93A mice, leading us to hypothesize the presence of a compensatory mechanism. Trained WT and SOD1-G93A mice exhibited a higher expression of the mitochondrial complex V subunit ATPB in the TA and a larger mitochondrial area in muscle fibers, and basically, the biogenesis of mitochondria and their content is linked to muscular strength (Lundby and Jacobs, 2016; MacInnis and Gibala, 2017; Distefano and Goodpaster, 2018).

In line with the early onset, the disease progressed faster in trained transgenic animals, as confirmed by the immunopathological and molecular analyses carried out at the end of training (91 days of age). Our results revealed massive NMJ dismantling and loss of motor neurons in the spinal cord, suggesting that training brought forward the pathological stage by approximately 30 days since 91-day-old trained SOD1-G93A mice resembled the phenotype of 120-day-old sedentary SOD1-G93A mice (Scaricamazza et al., 2020). In contrast to our finding, Carreras et al. (2010) concluded that intense endurance training did not worsen motor neuron loss in SOD1-G93A mice; however, their training protocol (number of training days, time, speed, and slope) was milder than ours. Noteworthy, it has been demonstrated that moderate-intensity aerobic exercise protected ALS-SOD1 mice from motor neuron loss, delaying the onset of motor symptoms and extending lifespan (Kirkinezos et al., 2003; Carreras et al., 2010). Furthermore, Ferri et al. (2019) reported that moderate-intensity aerobic exercise has a beneficial effect on ALS patients, improving their aerobic fitness and maintaining their physical functions.

We saw that the neuromuscular damage in SOD1-G93A mice, induced by training and characterized by NMJ dismantling and spinal motor neuron loss, came with neuroinflammation in both peripheral and central nervous systems. Moreover, we detected increased Wallerian degeneration with the infiltration of macrophages in the sciatic nerve and the activation of microglia and astrocytes in the spinal cord. Similarly, Kassa et al. (2017), using a milder protocol of endurance training, observed enhanced microglia activation in SOD1-G93A mice, even though the number of motor neurons and the onset of the disease were comparable to the sedentary controls.

Recent findings have highlighted the fact that exercise has anti-inflammatory effects on the whole body. In the nervous system, exercise prevents neuroinflammation by increasing the expression of neurotrophic factors and anti-inflammatory cytokines while reducing the levels of pro-inflammatory cytokines and activated microglia (Mee-Inta et al., 2019; Seo et al., 2019). Accordingly, in certain brain diseases, such as Alzheimer’s disease, Parkinson’s disease, and Huntington’s disease, exercise reduces neuroinflammation and improves prognosis (Svensson et al., 2015; Spielman et al., 2016; Seo et al., 2019). The studies have pointed out how the intensity of the exercise is crucial for neuroprotection (Zaychik et al., 2021). For instance, in a mouse model of multiple sclerosis (experimental autoimmune encephalomyelitis mice), high-intensity and regular training reduces autoimmune neuroinflammation by decreasing pro-inflammatory cytokines and microglial-derived ROS (Rizzo et al., 2021; Zaychik et al., 2021).

In light of these studies, it is imperative to highlight the controversial nature of ALS data, further considering that many studies, including this one, are based on preclinical models. Nevertheless, it is worth pointing out that while a physically active lifestyle is generally associated with a lower risk of brain diseases, addressing the role of PA in ALS requires closer scrutiny.

## 5 Conclusion

In SOD1-G93A mice, intense endurance exercise, administered before the onset of any ALS symptoms, anticipates the disease onset and worsens symptom progression by accelerating the pathological phenotype, suggesting that PA represents a risk factor in ALS. Moreover, intense endurance exercise in SOD1-G93A mice affects the entire skeletal muscle-motor neuron axis. This exercise protocol alters the muscular component by increasing the metabolic shift, induces the dismantling of the NMJ, generates inflammation in the peripheral nerve by altering its morphology, and increases the loss of motor neurons from the spinal cord ventral horns. In the present study, we also corroborated the theory about the important role of the interplay between skeletal muscle and motor neurons in the development of ALS disease. The so-called “dying-back hypothesis” proposes that a retrograde signaling cascade contributes to motor neuron degeneration. Here, we demonstrated that exacerbated damage to skeletal muscle through intense endurance exercise speeds up the denervation process and, thus, motor neuron loss. Although the intervention we proposed had a detrimental effect, it also suggests the pivotal role of skeletal muscle as a target in therapeutic strategies. Indeed, an intervention aimed at addressing the decline in muscle health has the potential to counteract the dying-back process and provide a protective shield for motor neurons. The physiological characteristics and accessibility of muscle tissue further enhance its appeal as a therapeutic target.

## Data Availability

The original contributions presented in the study are included in the article/Supplementary Material; further inquiries can be directed to the corresponding author.
