# Supplementary material for: Endurance exercise has a negative impact on the onset of SOD1-G93A ALS in female mice and affects the entire skeletal muscle-motor neuron axis
Source: Front Pharmacol. 2024 Mar 25;15:1360099. doi: 10.3389/fphar.2024.1360099 (PMC10999529; doi:10.3389/fphar.2024.1360099)
Supplement: Supplementary file 1 [file DataSheet1.docx]

Supplementary Material

##
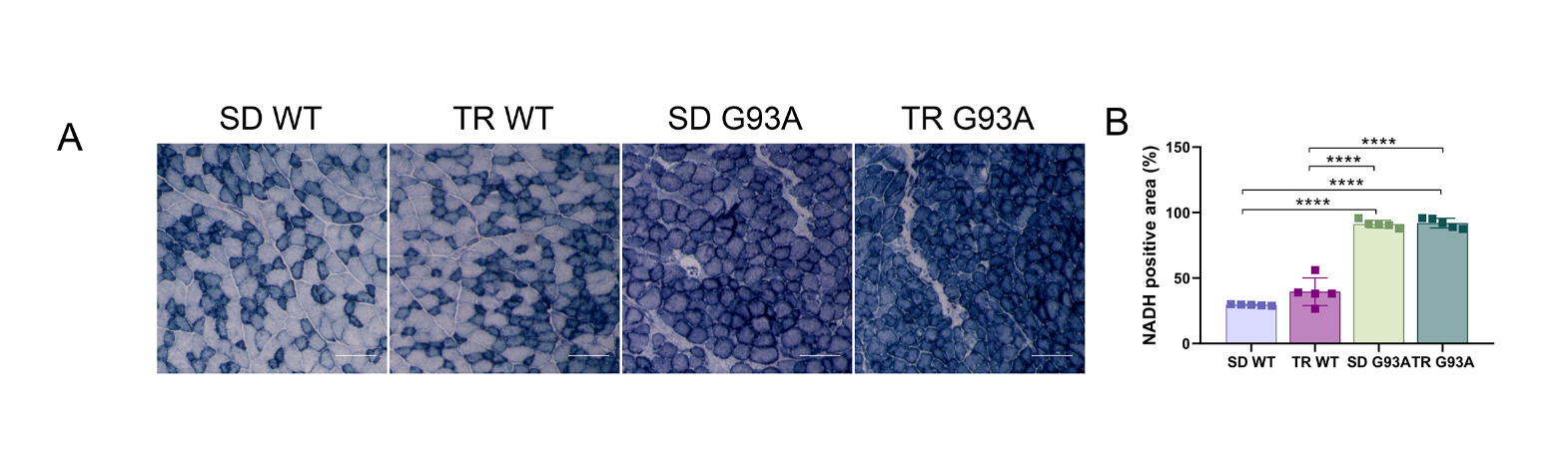
Supplementary Figures


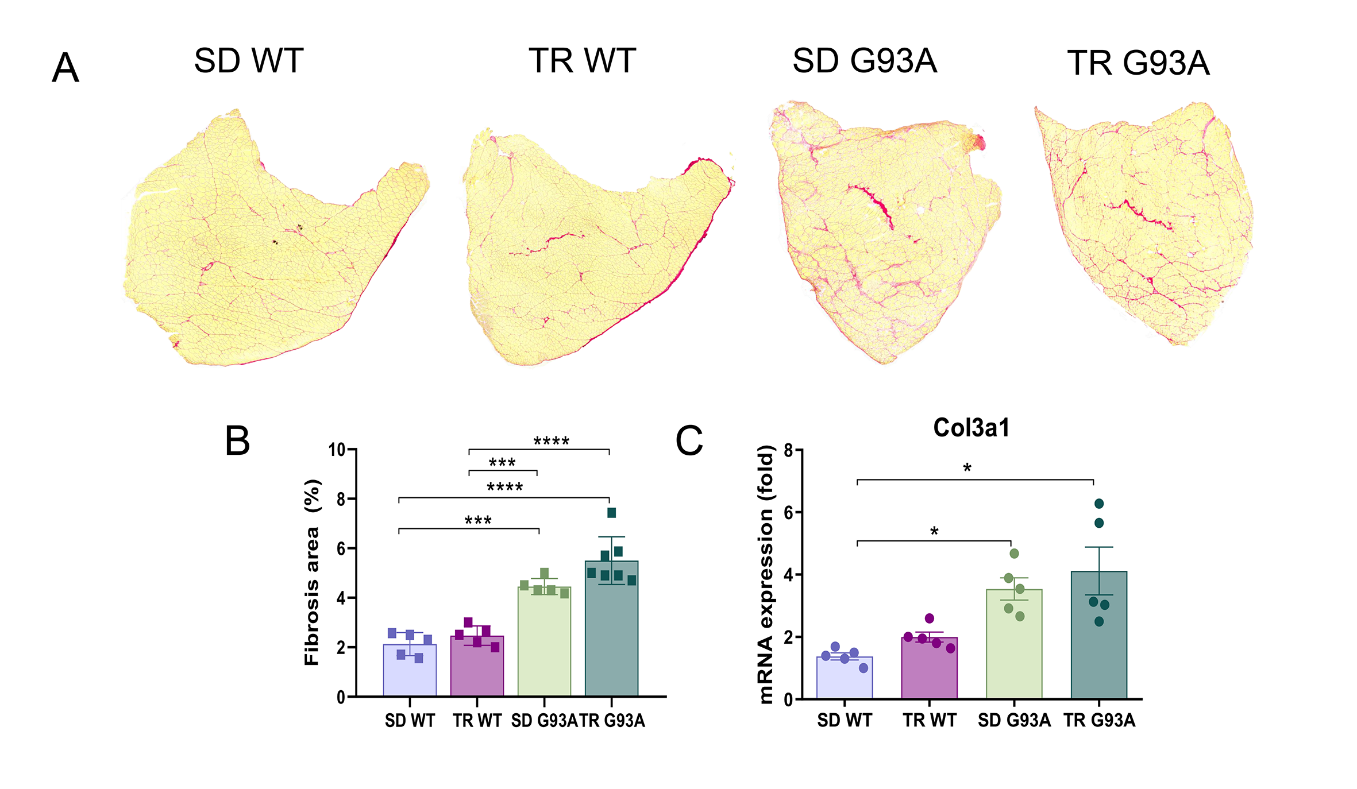
**Supplementary Figure 1.** A) Representative in situ NADH‐tetrazolium reductase activity staining in cross sections of TA muscle of 91-day-old sedentary and trained wild type and SOD1-G93A mice (SD WT, TR WT, SD G93A, TR G93A). Scale bar, 200 μm. B) Graphical representation of the percentage of NADH‐positive area. Data are presented as means ± SEM. ****P < 0.0001. P values were obtained using parametric two‐way ANOVA with Tukey post-hoc test. n ≥ 5 per group.

**Supplementary Figure 2.** A) Picro-Sirius red staining for fibrosis (red) in TA of 91-day-old sedentary and trained wild type and SOD1^G93A^ mice (SD WT, TR WT, SD G93A, TR G93A) and (B) graphical representation of the percentage of the fibrotic area. Data are presented as means ± SEM. ***P<0.001, ****P<0.0001. C) Expression level of mRNA encoding Col3a1 on TA of 91-day-old sedentary and trained wild type and SOD1^G93A^ mice (SD WT, TR WT, SD G93A, TR G93A). Data are presented as means ± SEM. *P<0.05, **P<0.01, ****P<0.0001. P values were obtained using parametric two‐way ANOVA with Tukey post-hoc test. n ≥ 5 per group.
